# Facile synthesis of flower shaped magnesium ferrite (MgFe_2_O_4_) impregnated mesoporous ordered silica foam and application for arsenic removal from water

**DOI:** 10.1038/s41598-023-48327-7

**Published:** 2023-12-07

**Authors:** Md. Jamal Uddin, Yeon-Koo Jeong

**Affiliations:** 1 Department of Soil and Environmental Sciences, University of Barishal, Barishal, 8254 Bangladesh; 2 Department of Environmental Engineering, Kumoh National Institute of Technology, 61 Daehak-ro, Gumi, Gyeongbuk 39177 Republic of Korea

**Keywords:** Nanoscale materials, Environmental, health and safety issues

## Abstract

Magnesium ferrite (MF_0.33_) impregnated flower-shaped mesoporous ordered silica foam (MOSF) was successfully synthesized in present study. MOSF was added with precursor solution of MF_0.33_ during MF_0.33_ synthesis which soaked the materials and further chemical changes occurred inside the pore. Therefore, no additional synthesis process was required for magnesium ferrite impregnated mesoporous ordered silica foam (MF_0.33_-MOSF) synthesis. MF_0.33_-MOSF showed higher morphological properties compared to other magnesium ferrite modified nanomaterials and adsorbed arsenic III [As(III)] and arsenic V [As(V)] 42.80 and 39.73 mg/g respectively. These were higher than those of other Fe-modified adsorbents at pH 7. As MOSF has no adsorption capacity, MF_0.33_ played key role to adsorb arsenic by MF_0.33_-MOSF. Data showed that MF_0.33_-MOSF contain about 2.5 times lower Fe and Mg than pure MF_0.33_ which was affected the arsenic adsorption capacity by MF_0.33_-MOSF. Adsorption results best fitted with Freundlich isotherm model. The possible mechanism of arsenic adsorption might be chemisorption by electrostatic attraction and inner or outer-sphere surface complex formation.

## Introduction

Arsenic toxicity is a well-known global problem affecting millions of people each year. Naturally arsenic comes from ground water. The use of ground water as drinking and irrigation purpose causes arsenic transfer to human health. Arsenic can cause several health dieses like keratosis, hyperkeratosis, melanosis, leucomelanosis, dorsum, and even cancer^1^. Governments are investing a lot of budgets for removal of arsenic from water. Among different technologies, adsorption is an efficient technology for arsenic removal from water^2^. Research data proved that iron-based adsorbents performed well over other adsorbents in arsenic removal^3^. However, modification through impregnation, coating and functionalization of iron adsorbents with porous material improved adsorbent characteristics which enhanced As removal efficiency^4–7^.

Different kinds of porous materials have been used for water treatment solely or as a composite with other material so far. Sand, clay, zeolite, activated carbon and silica are typical porous materials used for arsenic removal from water through adsorption process^8,9^. There were different advantages for using porous material as support or carrier material. Firstly, porous materials can reduce the aggregation of nanomaterials during adsorption process. This phenomenon decreases surface area and active sites of the adsorbent. Secondly, selectivity and catalytic activity of adsorbents for pollutant adsorption can be affected by the textural properties of the carrier material. Pollutants can form a thin layer on the pore channels or walls and be attached swiftly. Thirdly, encapsulation and impregnation of active nanomaterials into the pore and cavities of porous material increases the stability and dispersion of nanomaterials in catalysis process. Finally, for more flexibility, pore dimensions, morphology and structure of the porous materials can be designed as per requirement of the research. Activated carbon, zeolite, activated alumina can show upper three advantages but the final advantage is the specialty for mesoporous silica material which made this material more efficient over others^10^.

Porous silica material were considered as ideal material in different application because of exceptionally large surface area, thermal and chemical stability, high pore volume, low density, high selectivity and flexibility which can be modified using different organic and inorganic functional groups^10,11^. Among different type of porous silica materials, macro and mesoporous silica foam (MOSF) was found most suitable for pollutant removal from water through adsorption. Yang et al.^12^ synthesized lanthanum coated MOSF which removed 96% phosphorus within 30 min. They further modified MOSF with γ-Fe_2_O_3_ nanoparticles and applied for As(III) and As(V) removal from water. Data revealed that maximum 34.8% γ-Fe_2_O_3_ nanoparticles dispersed on pore walls of MOSF which adsorbed 248 and 320 mg/g As(III) and As(V) [Initial conc. of As(III) and As(V) was 560 and 600 mg/l] respectively^13^.

Iron was found most efficient element for arsenic removal through adsorption process^1^. Among Fe-based adsorbents, spinel ferrites exhibited exceptional performance in pollutant removal during water treatment. However, researchers found health toxicity because of cobalt ferrites. But there were no data found on magnesium ferrite (MgFe_2_O_4_) related toxicity in literature. In addition, magnesium plays essential functional activity inside human body. Besides that, among all spinel ferrites, magnesium ferrite showed better performance in pollutant removal through adsorption because of easy availability, safe handling, large surface area, tunable porosity and economically viable properties^2,14^. However, nanomaterials have some negative properties during adsorption such as aggregation and separation. Jung et al.^15^ showed that MgFe_2_O_4_ /Biochar composite adsorbed higher amount of phosphate than MgFe_2_O_4_ nanoparticles. Karthikeyan et al.^16^ also reported that modified MgFe_2_O_4_ was performed more efficiently for toxic ions removal than single MgFe_2_O_4_ nanoparticles. Based on the literature data, it was found that the modified material showed more stable morphological properties. In addition, modification increased the adsorptive site and influenced on pH_PZC_ that removed more toxic ions from solution. Furthermore, modification of magnesium ferrite not only increased adsorption capacity but also increased the stability and pollutant selectivity during adsorption process^15–17^. Considering the exceptional properties of MOSF in literature, present research was structured to develop a new composite material that might remove pollutants from water efficiently^10^.

Tile date, there was no literature found based on MgFe_2_O_4_ modified MOSF composite material. Very few research data were found on adsorptive arsenic removal from water using magnesium ferrite nanomaterials^14,18,19^. In addition, MOSF might increase MgFe_2_O_4_ nanomaterials stability and adsorption capacity which was the main objective of this research^12,13^. Therefore, considering efficiency and economic value, a simple Solvothermal method was followed for magnesium ferrite impregnated MOSF synthesis in present study and applied for arsenic removal from water.

## Materials and methods

All the chemicals and reagents used in present study were ACS-grade and supplied by Daihan Scientific, Republic of Korea. MgFe_2_O_4_ nanomaterial was synthesized by following a method with major modifications in different experimental factors^19,20^. To follow the ratio of Fe:Mg = 0.67:0.33, appropriate amount of anhydrous ferric chloride (FeCl_3_) and magnesium chloride (MgCl_2_) were dissolved in 70 ml ethanol (C_2_H_6_O). After that, 10 ml of just prepared 2.03 M sodium hydroxide-ethanol (NaOH-C_2_H_6_O) mixture added to the solution. To make a homogenized solution, the mixed solution was taken under a Sonicator for 1 h at 25 ± 5 °C temperature. Sonicator probe height, amplitude and temperature were carefully maintained for all runs. The homogenized solution mixture was taken into a Teflon-line autoclave made of stainless steel and closed it tightly. Then the autoclave was kept in a convective oven for 8 h at 200 °C temperature. The autoclave was naturally cooled to room temperature. After that, the mixture was transferred into a 100 ml beaker and washed using deionized water with the help of magnetic stirrer. The washing process was continued until the mixture was free from sodium chloride. The final precipitate was transferred to a ceramic cup and taken into the oven for 12 h at 80 °C temperature. After that time period, the ceramic cup was kept in a desiccator carefully and naturally cooled. Next, the dried materials were grounded using a ceramic mortar where a dark brown finer material produced. Finally, the material was transferred into a dark glass bottle and preserved for characterization and adsorption experiments.

The method for mesoporous silica foam synthesis under present study was followed the method described by Wang et al.^21^ with some minor changes. At first, 1 g of P123 was measured in a beaker and kept it in a water bath having 35 °C. Then, 30 ml of 0.40 M sodium sulphate (Na_2_SO_4_) solution was added and stirred continuously. Subsequently, 31 ml of 0.02 M sodium acetate-acetic acid buffer solution was added under continuous stirring. The mixture was stirred until a homogenous mixture solution formed. Next, 1.48 ml of tetramethyl orthosilicate (TMOS) solution was added to the solution mixture and stirred for 5 min further. The beaker containing solution mixture was kept in an incubator under 35 °C. After 24 h of incubation, the mixture was sealed in a teflon-lined-autoclave and leaved it in the oven for another 24 h at 100 °C. After that, the autoclave was naturally cooled and the mixture was washed with water until it became sodium free. Finally, the white precipitate was air dried and calcined at 550 ˚C temperature for 5 h in a muffle furnace. The white colored material then was cooled in a desiccator and preserved for further analysis.

The impregnation in this study was very simple and no further chemicals were required. Appropriate amount of anhydrous FeCl_3_ and MgCl_2_ were mixed with synthesized MOSF in 70 ml ethanol under sonication. After 30 min of sonication, freshly prepared NaOH solution (10 ml) was added to the mixture drop wise under continuous sonication. After 1 h of sonication, the mixture was sealed in the teflon-lined-autoclave and heated at 200 °C temperature for 8 h in the oven. Next, the naturally cooled mixture was washed with deionized water for several times until the mixture was become sodium chloride (NaCl) free with the help of 2 min continuous sonication and 10 min magnetic stirring. The drying and grinding process of the washed material was same as magnesium ferrite synthesis. The magnesium ferrite nanomaterial having molar ratio of magnesium and iron 0.33:0.67 was expressed as MF_0.33_ and this was incorporated into MOSF. The end material was used for characterization and adsorption process.

### Characterization

Powder X-ray diffraction (XRD) pattern (Rigaku Japan, using filtered Cu Kα radiation) of the synthesized material was determined for crystal phase identification. The average crystallographic size of synthesized material was calculated from the most intense peaks on XRD spectra using Scherrer’s equation. The elemental composition and morphology was investigated using field emission electron microscopy (SEM; MAIA3 TESCAN) functioning with an energy dispersive X-ray (EDS). Chemical composition and bonding nature were proved by Fourier transform-infrared spectroscopy (FT-IR; BRUKER). Magnetic properties of the synthesized materials were analyzed by vibrating sample magnetometer (VSM, Lake Shore Cryotronics, Inc.). N_2_ adsorption–desorption isotherm was recorded from micromeritics. Average pore size, shape, volume and specific surface area were obtained using Barrett-Joyner-Halenda (BJH) and Brunauer–Emmett–Teller (BET) method.

### Adsorption experiments

To observe the adsorption efficiency of MF_0.33_-MOSF, nine sets (1, 5, 10, 25, 50, 100, 150, 200 and 300 mg/l) of As(III) and eight sets (1, 5, 10, 20, 40, 60, 100 and 130 mg/l) of As(V) concentration were prepared from stock solution (1000 mg/l). 1.0 g/l adsorbent dose was applied. pH values were measured before and after adding adsorbent. Dilute hydrochloric acid (HCl) and NaOH were used to adjust pH 7 after adsorbents addition. The conical flasks containing arsenic solution and adsorbent were kept in a shaking incubator for 12 h. The incubator was operated at 300 ± 5 rpm and temperature was controlled at 25 °C. Next, the mixture solutions were centrifuged for 1 h at 4000 rpm to separate solids. Then, approximately 20 ml clear solution was transferred to plastic tube. To avoid further oxidation, 1 drop of concentrated HCl was added to each solution tube and stored. These solutions were used to determine equilibrium As(III) and As(V) concentration using inductively coupled plasma optical emission spectroscopy (ICP-OES). Adsorption capacity of synthesized MF_0.33_-MOSF nanomaterial was computed according to the equation given below^22^:

1 $${\text{Arsenic}}\;{\text{ adsorption}}\;{\text{ capacity}},\;q_{e} \; = \;\frac{V }{{m }} \times \left( {C_{0} - C_{e} } \right)$$

where, C_0_ (mg/l) is the initial concentration of As(III) and As(V), C_e_ (mg/l) is the equilibrium concentration of As(III) and As (V), V (l) for volume, m (g) for adsorbent dose and q_e_ (mg/g) for adsorption capacity at equilibrium respectively.

To quantify maximum arsenic adsorption capacity and possible arsenic adsorption mechanism by synthesized MF_0.33_ impregnated MOSF, two adsorption isotherm models have been applied named Freundlich (Freundlich, 1906) adsorption isotherm model and Langmuir (Langmuir, 1916) adsorption isotherm model.

The Freundlich adsorption isotherm model can be expressed as follows:

2 $$q_{e} = K_{f} C_{e}^{\frac{1}{n}}$$

where, q_e_ is the arsenic adsorption capacity by adsorbent at equilibrium (mg/g), C_e_ is the equilibrium arsenic concentration in water (mg/l), K_f_ (mg^1–1/n^l^1/n^g^−1^) and n is the Freundlich empirical constants which are related to the maximum arsenic adsorption and the 1/n is the heterogeneity factor of the nanoadsorbent representing the strength of adsorption. The value of n should be lie between 1 and 10 for favorable adsorption process^23^.

The Langmuir adsorption isotherm model can be expressed as follows:

3 $$q_{e} = \frac{{q_{m} K_{L} C_{e} }}{{\left( {1 + K_{L} C_{e} } \right)}}$$

where, K_L_ (l/mg) designated as Langmuir adsorption constant, C_e_ is the equilibrium arsenic concentration (mg/l), q_e_ (mg/g) is the arsenic adsorption capacity at equilibrium and q_m_ (mg/g) represents maximum adsorption capacity of As(III) and As(V) on MF_0.33_-MOSF. The affinity of arsenic toward binding site is related with K_L_.

### Determination of pH_PZC_

Point of zero charge (pH_PZC_) of MF_0.33_-MOSF nanomaterial was identified through pH Drift method^24^. In short, based on pH, 10 different sets of vial (pH 3–12) were prepared by adding 15 ml NaCl solution having 0.1 M concentration. pH 3–12 was made by using HCl or NaOH solutions (for pH ˂ 7, HCl and for pH ˃ 7, NaOH). Subsequently, 0.015 g of MF_0.33_-MOSF nanomaterial was added to each vial. The vials were stirred 4 h using a magnetic stirrer and kept it in a temperature (25 °C) controlled incubator for 24 h. The final pH of each vials were measured and an initial pH vs pH change (ΔpH) graph was made. The line pH-ΔpH crossed the point of pH zero line is the pH_PZC_ of the material.

### Ethical approval

This is an original research and has not been submitted elsewhere at the same time. The whole research compiled in a single manuscript which submitted to *Scientific Reports*. A new material named MOSF modified MF was first time prepared and applied for arsenic removal by the authors. Before submission, the manuscript was checked for plagiarism using Turnitin software.

## Results and discussion

### Characteristics of magnesium ferrite impregnated MOSF

#### XRD

**Figure 1:**
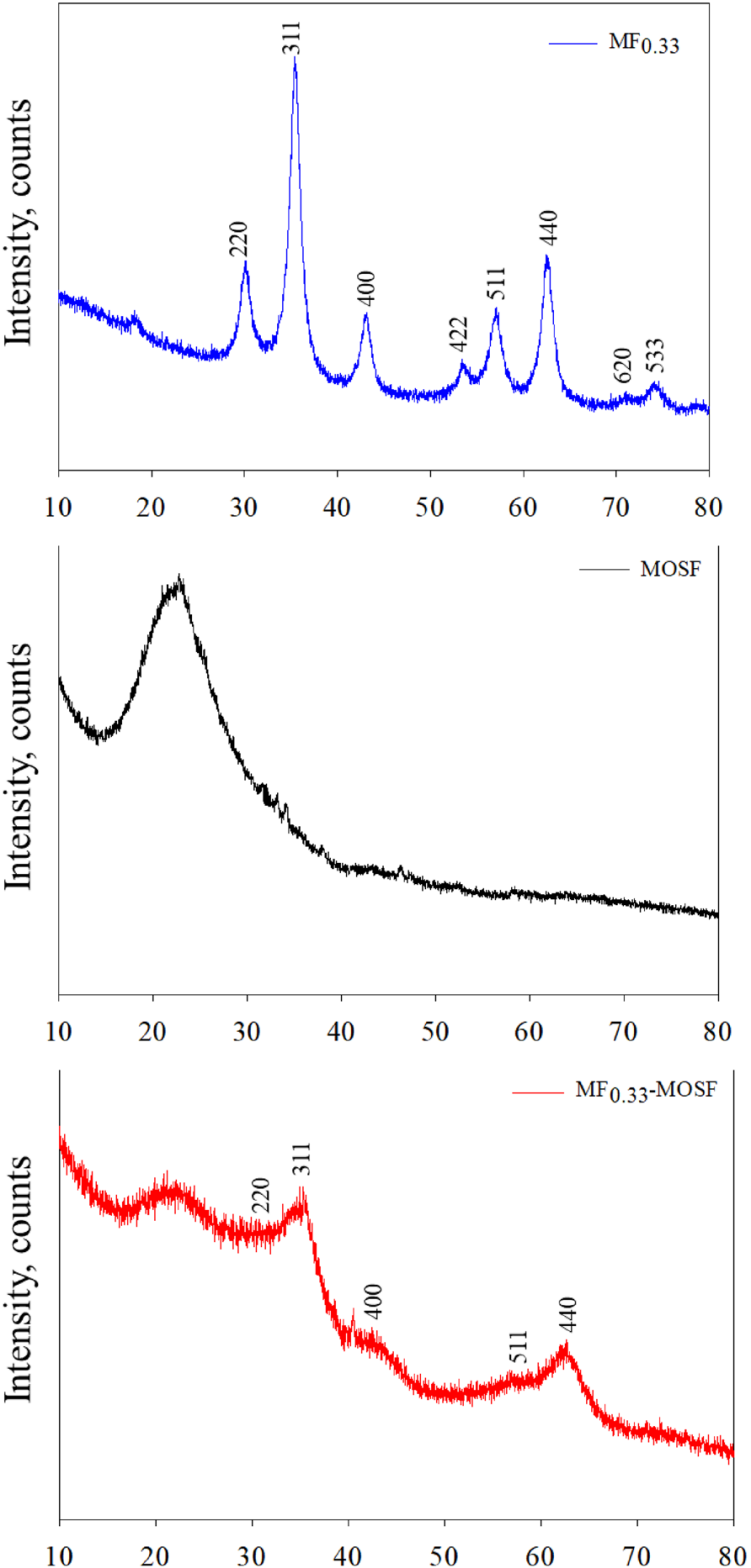
XRD spectra of synthesized nanomaterials.

The XRD spectra of synthesized MF_0.33_, MOSF and MF_0.33_-MOSF material at 2θ scale ranging from 10° to 80° were shown in Fig. 1. For MOSF, only one peak was found at 2θ = 22° on the XRD spectra which was representative for the formation of amorphous silica^25^. The peak pattern found in MF_0.33_ demonstrated successful synthesis of single phase [S/M (PDF-2 release 2020 RDB)] cubic shape magnesium ferrite spinels which belongs to space group Fd3m (227:Fd-3 m:2; a = 8.39913 Å). Figure 1 showed diffraction peaks at 2θ = 18.31 (111), 30.09 (220), 35.40 (311), 43.05 (400), 53.35 (422), 56.95 (511), 62.53 (440), 71.00 (620) and 74.10 (533) which was properly fitted with inorganic crystal structure database (ICSD-01-076-9733). MF_0.33_-MOSF produced 6 peaks on the XRD spectra. The peak at 2θ = 22° confirmed the amorphous silica and 2θ = 31.10 (220), 35.30 (311), 43.43 (400), 56.46 (511) and 62.64 (440) were attributed to the spinel magnesium ferrite nanomaterial, which was appropriately fitted with standard XRD planes for cubic-spinel shaped MgFe_2_O_4_ nanomaterial card number ICSD-01-076-9733.

Therefore, based on the comparison results, the new synthesized material contained both amorphous silica phase and magnesium ferrite spinels. These data also confirmed that magnesium ferrite crystals were well established in MOSF structure. Intense peaks of the XRD plane were used to calculate average crystallite size according to Debye–Scherrer’s formula^26,27^.

According to the Debye–Scherrer’s formula,

4 $${\text{Crystalline }}\;{\text{size}},\;{\text{ D }}\; = \; \, \left( {K\lambda / \, \beta {\text{ Cos}}\theta } \right)$$

where, D (nm) average crystalline size, *K* (*K* = 0.89) is considered as Scherer’s constant, *λ* (Å) represents the applied wavelength of X-ray, β (radian) represents as the full width at half maximum (FWHM) of the diffraction peak and *θ* represents diffraction angle produced by the peak.

The calculated average crystallite size was found 5.85, 0.75 and 1.84 nm for MF_0.33_, MOSF and MF_0.33_-MOSF respectively. There were no other peaks found on the XRD plane which confirmed that the material synthesized under present research is in single phase state with high purity.

#### N_2_ adsorption–desorption isotherms

**Figure 2:**
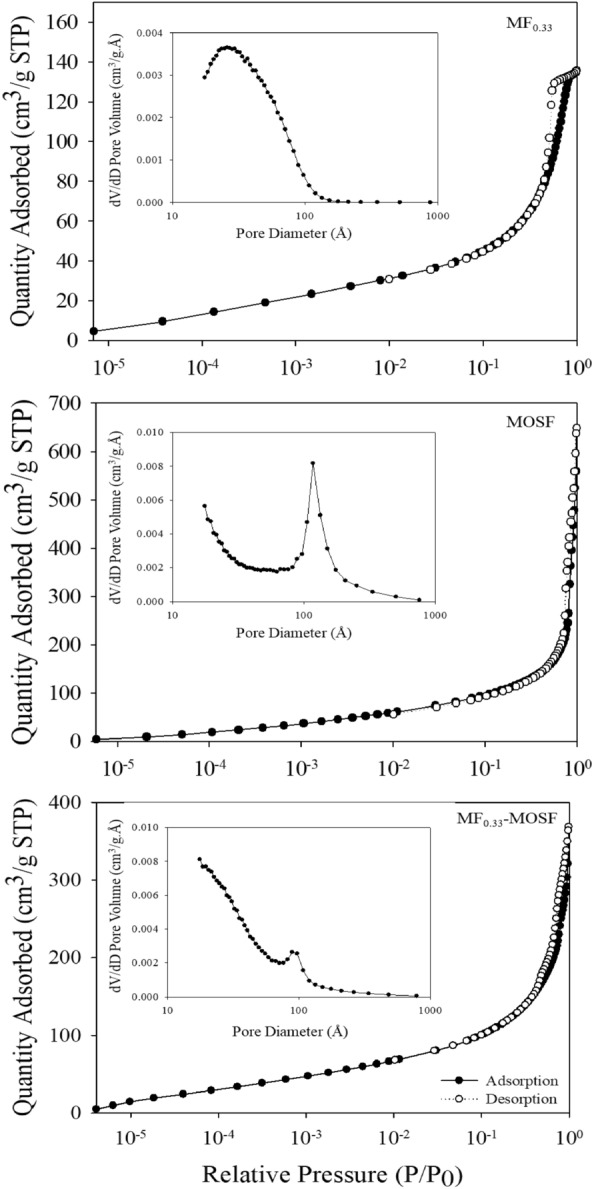
N_2_ adsorption–desorption isotherms of MF_0.33_, MOSF and MF_0.33_-MOSF.

Specific surface area, average pore volume and pore size of MF_0.33_, MOSF and MF_0.33_-MOSF were measured through N_2_ adsorption–desorption isotherm analysis, as represented in Fig. 2. Comparing with international union of pure and applied chemistry (IUPAC) provided classification of hysteresis loop, the pattern displayed in N_2_ adsorption–desorption curves were matched with single mode IV which belongs to H1 hysteresis loop. Based on IUPAC classification, this type of hysteresis mode corresponding to the presence of abundant mesoporous pores^28^. The Barrett-Joyner-Halenda (BJH) pore size distribution pattern revealed average pore diameter of MF_0.33_, MOSF and MF_0.33_-MOSF were 4.17, 11.41 and 6.05 nm respectively (Table S1). MF_0.33_ impregnation in MOSF decreased the pore diameter, which might be resulted from the incorporation of MF_0.33_ particles in pore of MOSF. The Brunauer–Emmett–Teller (BET) total pore volume and specific surface area of mesoporous MF_0.33_, MOSF and MF_0.33_-MOSF nanomaterials were 0.2083, 0.8012, 0.4684 cm^3^/g and 200.36, 412.86, 427.04 m^2^/g respectively. Owing to impregnation, BET surface area was increased, which indicated that the particles size of MF_0.33_-MOSF was decreased after impregnation. The average nanoparticle size found by BET analysis (29.95, 14.53 and 14.05 for MF_0.33_, MOSF and MF_0.33_-MOSF respectively) also supported the increment of surface area. The decrease in total pore volume in impregnated material might be also resulted from the impregnated magnesium ferrite in MOSF. Therefore, the MF_0.33_-MOSF nanomaterial showed exceptionally large surface area, high pore volume and mesoporous pore size that would be an efficient adsorbent for pollutant removal from water.

#### FTIR

**Figure 3:**
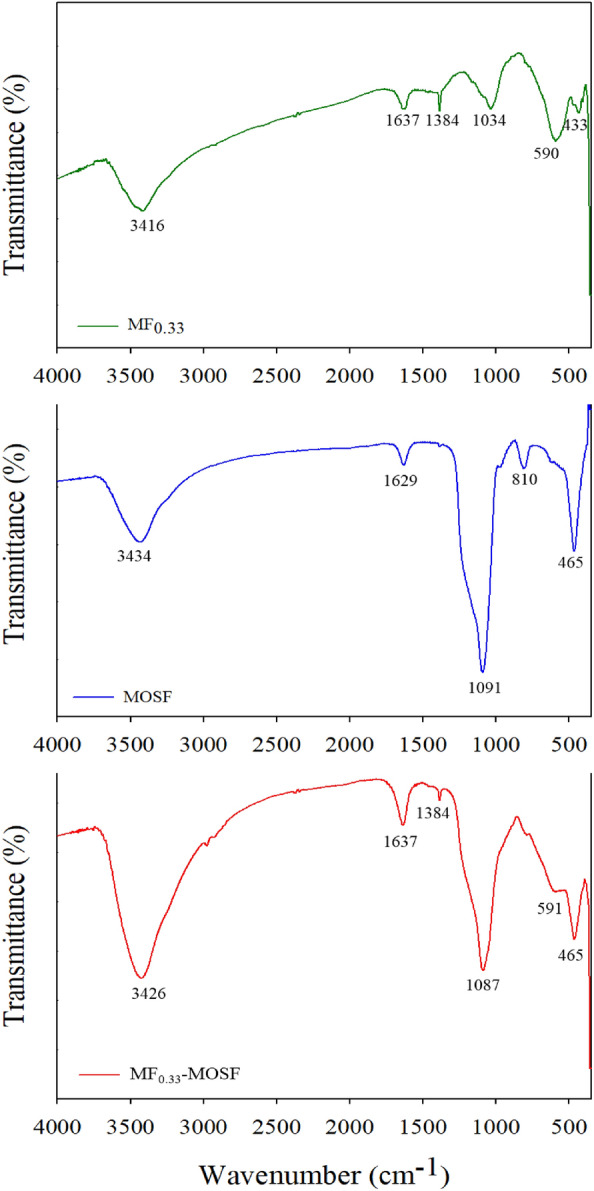
FTIR spectra of MF_0.33_, MOSF and MF_0.33_-MOSF nanomaterials.

To recognize the bonding types, nature and functional groups of the MF_0.33_, MOSF and MF_0.33_-MOSF nanomaterials, FTIR analysis was performed and results showed in Fig. 3. The IR data was collected in range of 400–4500/cm of wavenumber. The distinctive adsorption peaks of MF_0.33_ nanomaterial were appeared at 3416, 1637, 1384, 1034, 590 and 433/cm region were identical to the literature data for magnesium ferrite nanomaterial^14,29^. The broader peak appeared at 3416/cm and the sharp peak turned up at 1637/cm region were associated with stretching and bending vibrations of –O–H groups and surface adsorbed water (H_2_O) by hydrogen bond on MF_0.33_ surface. The other sharp peaks produced at 1384/cm and 1034/cm region of the spectra were assigned to deformation and bending vibrations of metal hydroxide (M-OH^−^), which coordinated to Fe^3+^ or Mg^2+^. The intense peaks appeared at 590/cm and 433/cm were related to the innate vibrations of octahedral and tetrahedral metal oxides (M–O)^29^. For MOSF, the peak at 3434/cm was attributed to stretching vibration of –OH on the MOSF surface. The bending vibration of the adsorbed water on the surface absorbed the light 1629/cm. Additionally, the peak at 1091/cm and 810/cm were attributed to asymmetric and symmetric stretching vibrations of Si–O–Si bond. Furthermore, the peak produced at 465/cm was assigned to the structural SiO_4_ tetrahedra^30,31^. The FTIR spectra for MF_0.33_-MOSF nanomaterial showed almost all the chemical bonds observed in MF_0.33_ and MOSF. These results also confirmed the successful impregnation of MF_0.33_ in MOSF.

#### SEM–EDS

**Figure 4:**
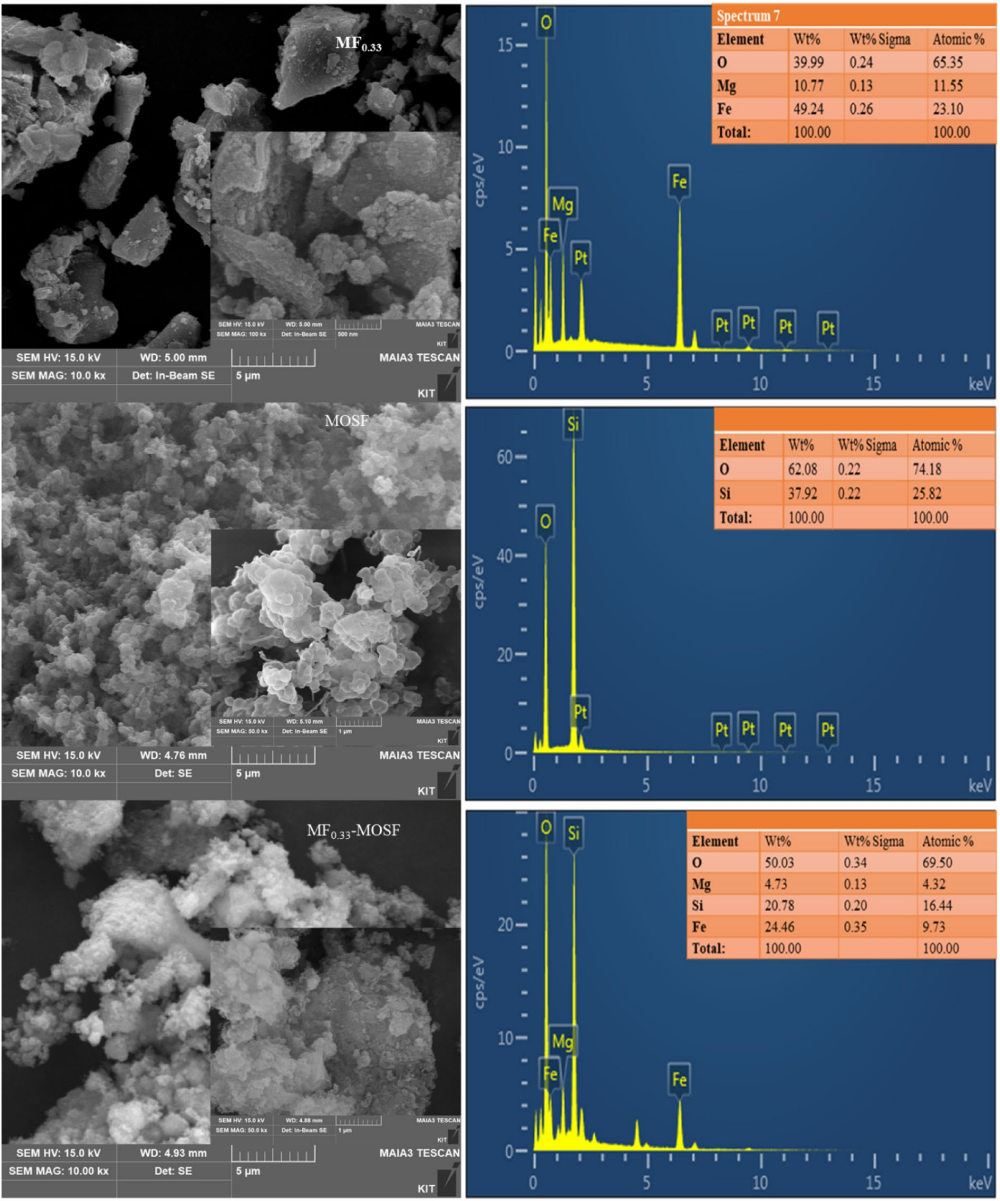
SEM–EDS of MF_0.33_, MOSF and MF_0.33_-MOSF nanomaterial.

Surface morphology of MF_0.33_, MOSF and MF_0.33_-MOSF nanomaterials were observed through SEM analysis (Fig. 4). The synthesis method of MF_0.33_ and MF_0.33_-MOSF nanomaterial was divided into four steps which were homogenization, thermal treatment, washing and drying. The synthesized materials after thermal treatment remain as finer particles which suspended rapidly in water during washing of the material for NaCl removal. However, the nanomaterials formed small agglomerates after 12 h heating in the oven for drying. Therefore, naturally cooled agglomerates were taken into a ceramic mortar and grounded to finer particles. The SEM image of MOSF nanomaterials was showed flower shape in appearance and there was no need to grind. Because of grounding, the agglomerates of MF_0.33_ were broken through the boundary line between the cubic spinel shape particles which were shown in Fig. 4. Although, the impregnated material showed similar agglomerates like MF_0.33_ but clear spinels could not be found in the impregnated materials like MF_0.33_ which might be due the growth of MF_0.33_ spinels inside the pores of flower shape MOSF nanomaterial. This also supported the data found in N_2_ adsorption–desorption isotherm analysis. Based on that data, the MOSF nanomaterial was found mesoporous having large pores. And it was also found that the size of MOSF particles were smaller than that of MF_0.33_. After impregnation, the pore size and pore volume of MOSF nanomaterial decreased and a new shape was found in MF_0.33_-MOSF image which has similarities with flower shape MOSF.

For the evaluation of elemental composition in each nanomaterial, EDS analysis has been done (Fig. 4). The EDS analysis of MF_0.33_-MOSF proved the presence of Si, Fe, Mg and O element, which came from MF_0.33_ and MOSF. The MF_0.33_ nanomaterial contains 23.10% of Fe and 11.55% (atomic weight base) of Mg. But after impregnation of MF_0.33_ in MOSF by 1:1 ratio, MF_0.33_-MOSF contain approximately 2.37 times lower Fe and 2.67 times lower Mg than MF_0.33_. This lower amount of Fe and Mg in MF_0.33_-MOSF might affect the adsorption capacity of the impregnated material. EDS result also showed that there were no other element presents in the synthesized material, which confirmed phase purity of the nanomaterials. These results confirmed that the phase pure MF_0.33_ impregnated MOSF nanomaterial had been successfully synthesized in present study.

#### VSM

**Figure 5:**
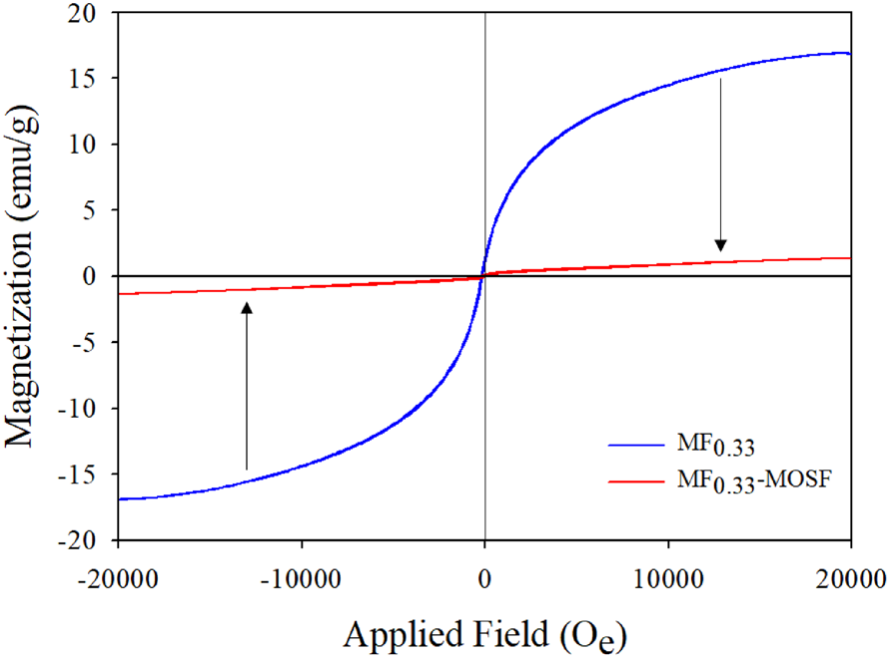
Magnetization curves of synthesized MF_0.33_ and MF_0.33_-MOSF nanomaterials at room temperature.

Magnetic properties of synthesized MF_0.33_ and MF_0.33_-MOSF nanomaterials were analyzed by vibrating sample magnetometer (VSM) at room temperature with an application of magnetism of − 20,000 to 20,000 Oersted (Oe) (Table S2). The magnetic saturation (Ms), retentivity (Mr) and coercivity (Hc) were found 16.90 emu/g, 1.30 emu/g, 30.15 Oe for pure MF_0.33_ and 1.36 emu/g, 0.14 emu/g, 153.14 Oe for MF_0.33_-MOSF from VSM data respectively. Pure MgFe_2_O_4_ phase showed S-shaped magnetization curve (Fig. 5), which indicated that MF_0.33_ was superparamagnetic material and can be separated easily by an external magnet after adsorption. Conversely, MOSF is a non-magnetic material. After impregnation of MF_0.33_ in MOSF, the new material showed week magnetic properties.

Magnetization saturation of the synthesized nanomaterials was calculated using the formula:

5 $${\text{Saturation }}\;{\text{magnetization}},{\text{ Ms }} = \, \upvarphi {\text{mS}}$$

where, mS is the saturation moment of a single particle and φ is the volume fraction, it is clear that Ms can be determined by volume fractions and intrinsic properties (saturation moment) of materials involved. Thus, low magnetism of MF_0.33_-MOSF can be attributed to low amount of incorporated MF_0.33_ in it^32^. The magnetic properties decreased after impregnation but magnetic properties of the MF_0.33_-MOSF could be tuned by controlling these two parameters.

Coercivity depends on particle size which will increase up to certain limit with decreasing particle size^33^. Based on BET data, particles size of MF_0.33_ was found 29.95 nm and after impregnation, particle size was 14.05 nm for MF_0.33_-MOSF. Therefore, coercivity increased in MF_0.33_-MOSF than MF_0.33_. The remnant magnetization (Mr) is the magnetization left after removing external magnetic field from a material. The Mr value was found 1.30 emu/g for pure MF_0.33_ and 0.14 emu/g for MF_0.33_ impregnated MOSF. Superparamagnetic MF_0.33_ showed 9.29 times higher Mr than MF_0.33_-MOSF. The ratio of Mr and Ms is called squareness which is important properties for ferromagnetic materials. Depending on processing, Mr/Ms ratio of commercial magnets varied in the range 0.88–0.96^34^. The squareness ratio were found 0.08 and 0.10 for MF_0.33_ and MF_0.33_-MOSF. Because of cubic spinel shape, MF_0.33_ has less Mr/Ms which increased impregnated material.

Zhou et al.^35^ found similar magnetization result when synthesized silver phosphate@magnesium ferrite [Ag_3_PO_4_@MgFe_2_O_4_] nanocomposites. The magnetization of pure MgFe_2_O_4_ material was found 15 emu/g which was decreased to 1.6 emu/g in Ag_3_PO_4_@MgFe_2_O_4_ (10%). They showed that non-magnetic Ag_3_PO_4_ attained good magnetic properties after composite formation with superparamagnetic MgFe_2_O_4_ that can be separated easily from water by a magnet^35^. Khafagy^32^ showed that magnetization of pure phase MgFe_2_O_4_ decreased (21.33–5.905 emu/g) when coating MgFe_2_O_4_ with polyaniline. They also found that coercivity decreased 88.66 Oe–81.60 Oe after coating because the particle size of pure MgFe_2_O_4_ (30–35 nm) increased (45 nm) after coating. Hoijang et al.^17^ synthesized silica-coated MgFe_2_O_4_ showing superparamagnetic properties. The magnetization of pure MgFe_2_O_4_ and silica-coated MgFe_2_O_4_ were found 37 and 27 emu/g. During synthesis, they added only 1 ml tetraethyl orthosilicate in a solution containing 200 mg of MgFe_2_O_4_. Because of higher amount of MgFe_2_O_4_, the magnetic properties was not decreased much. Therefore, magnetic properties of the nanomaterials synthesized in present study could be increased by changing MF_0.33_ and MOSF ratio.

#### Comparative study of morphological characteristics

Magnesium ferrite impregnated mesoporous ordered silica foam has been synthesized for the first time under present study. The modified magnesium ferrite nanomaterial showed exceptionally large surface area compared to other modified magnesium ferrite nanomaterials found in literature till date (Table S3). Tiwari and Kaur^36^, synthesized silica@magnesium ferrite [SiO_2_@MgFe_2_O_4_] material having higher surface area and pore volume than MF_0.33_-MOSF synthesized in this research. But the pore size of SiO_2_@MgFe_2_O_4_ material approximately 1.71 times lower than MF_0.33_-MOSF. In addition, complex synthesis process was involved in SiO_2_@MgFe_2_O_4_ synthesis. At first they synthesized SiO_2_ and MgFe_2_O_4_ nanomaterial. After that further synthesis process was involved for SiO_2_@MgFe_2_O_4_ synthesis^36^. Whereas, the synthesis of MF_0.33_-MOSF material was very much simple in present study. At first MOSF synthesized and then this MOSF mixed with other precursor solutions during MF_0.33_ synthesis. Therefore, no further synthesis steps were involved, which could save energy and cost of the research. So, present research successfully synthesized MF_0.33_-MOSF nanomaterial through a simple and cost effective way having large pore size, pore volume and surface area compared to other nanomaterials found in literature (Table S3).

### Arsenic adsorption capacity

#### Adsorption isotherm

**Figure 6:**
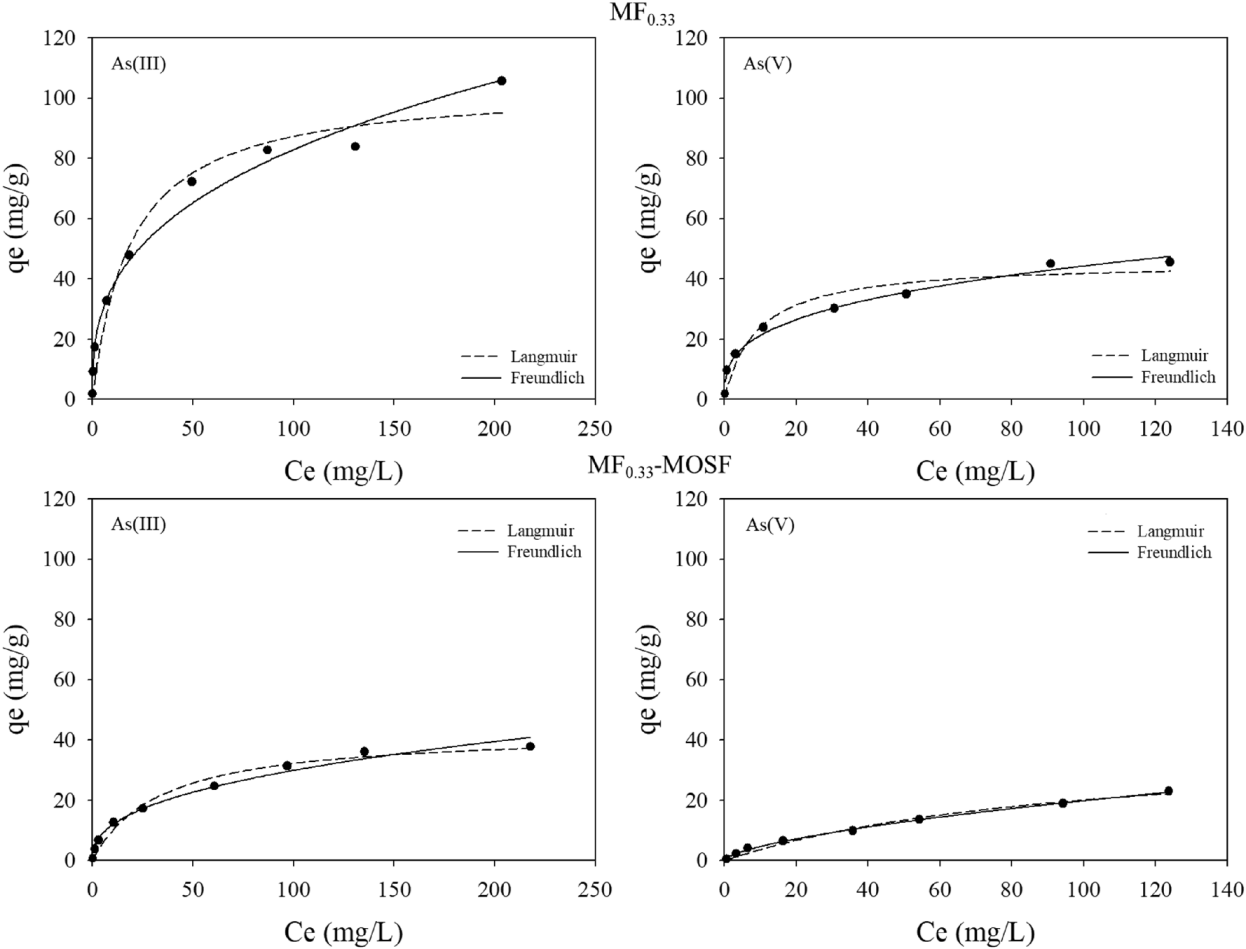
As(III) and As(V) adsorption isotherm of MF_0.33_ and MF_0.33_-MOSF nanomaterial. Conditions: pH = 7, Temperature = 25 °C, adsorbent dose = 1 g/l.

Table S4 showed the equilibrium arsenic adsorption isotherm results. The adsorption of both As(III) and As(V) species on synthesized MF_0.33_ and MF_0.33_-MOSF nanomaterial were tried to fit in Langmuir and Freundlich isotherm model (Fig. 6). The fitting results showed that adsorption data was best fitted on Freundlich model based on coefficient of correlation (r^2^) data. According to Foo and Hameed^37^, Langmuir adsorption isotherm is an equation based on monolayer adsorption of adsorbate onto adsorbent surface. Besides, Freundlich adsorption isotherm model is based on multilayer adsorption of adsorbate onto heterogeneous surface of the adsorbent which is not restricted to monolayer formation^37,38^. Therefore, multilayer adsorption of both arsenic species was taken place on MF_0.33_ and MF_0.33_-MOSF heterogeneous surface. The heterogeneity factor, 1/n can be found from Freundlich model. The value 0.1 ˂ 1/n ˂ 1 describes efficient adsorption of adsorbate on adsorbent surface^39^. In present study, the parameter 1/n calculated from Freundlich model were found 0.34, 0.32 by MF_0.33_ and 0.40, 0.63 by MF_0.33_-MOSF for As(III) and As(V) adsorption, which indicated that both species adsorbed easily on MF_0.33_ and MF_0.33_-MOSF surface^40^.

The maximum arsenic adsorption capacity of MF_0.33_ and MF_0.33_-MOSF nanomaterial was found in Langmuir isotherm model. Based on model results, MF_0.33_ and MF_0.33_-MOSF adsorbed 103.94, 42.80 mg/g of As(III) and 45.52, 39.73 mg/g of As(V) at pH 7 respectively. With increasing initial arsenic concentration, adsorption capacity was also increased. At equilibrium, MF_0.33_ nanomaterials adsorbed approximately 2.4 times higher amount of As(III) than MF_0.33_-MOSF. For As(V) adsorption, MF_0.33_ adsorbed slightly higher amount than MF_0.33_-MOSF. As the MOSF showed no arsenic adsorption capacity, MF_0.33_ was totally responsible for arsenic adsorption. Besides that, based on EDS results, MF_0.33_-MOSF contained 2.37 times lower Fe and 2.67 times lower Mg than MF_0.33_ when MF_0.33_-MOSF was synthesized in 1:1 ratio of MF_0.33_ and MOSF. Elemental analysis data by ICP-OES also showed that MF_0.33_-MOSF contain 2.47 times lower Fe and 2.42 times lower Mg than pure MF_0.33_. Therefore, the lower arsenic adsorption capacity was due to lower Fe and Mg content in MF_0.33_-MOSF nanomaterial, which could be enhanced by changing the MF_0.33_ and MOSF ratio.

#### Adsorption mechanism

**Figure 7:**
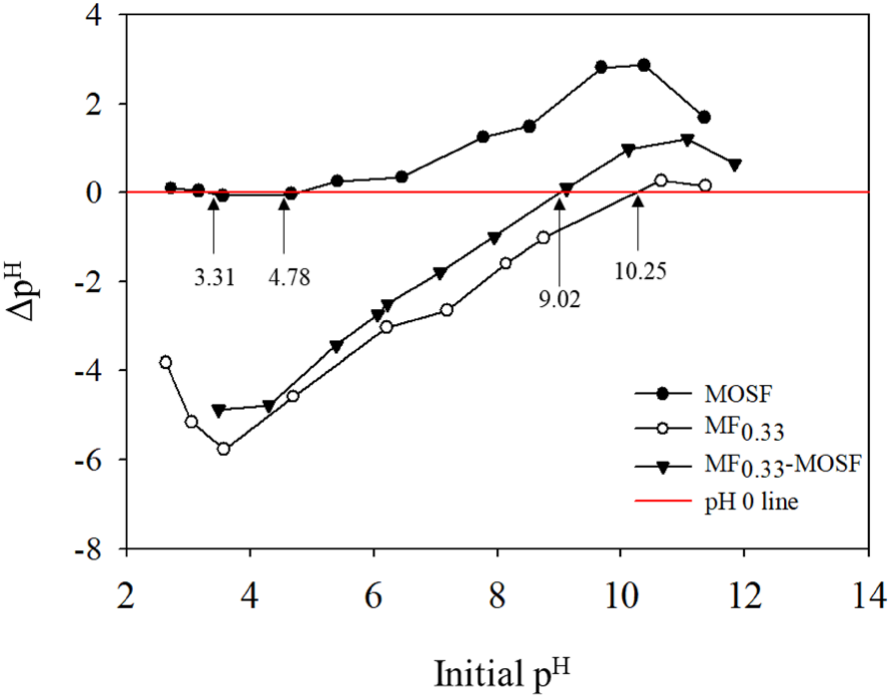
pH_PZC_ of MOSF, MF_0.33_ and MF_0.33_-MOSF nanomaterials.

Arsenic adsorption on MF_0.33_-MOSF nanomaterial can be discussed based on isotherm data and pH_PZC_ of the material. Adsorption results were well fitted to Freundlich isotherm model indicating that arsenic adsorption occurred on heterogeneous surface of MF_0.33_-MOSF nanomaterial. Therefore, possible mechanism of arsenic adsorption might be through physisorption and chemisorption. In addition, the pH_PZC_ of MF_0.33_-MOSF was found 9.02 which indicated that the nanomaterial surface was positively charged at pH 7 (pH ˂ pH_PZC_). The available forms of As(III) and As(V) under pH 7 are H_3_AsO_3_, H_2_AsO_4_^-^ and HAsO_4_^2-^ which might be easily adsorbed on MF_0.33_-MOSF surface through electrostatic attraction, ion exchange and complex formation. There were two pH_PZC_ (3.31 and 4.78) found for pure MOSF material (Fig. 7). Chrzanowska et al.^41^ found pH_PZC_ 4–5.6 of pure mesocellular silica foam. Derylo-Marczewska et al.^42^ found pH_PZC_ 4.93 of pure mesoporous silica foam which changed to 6.5 after protein adsorption. Brönsted acidity in silica surface is a well-known properties and pH_PZC_ of these materials was found at a range 2–3 which indicated that silica surface have positive charge (pH ˂ pH_PZC_) at very lower pH. In addition, after coating with magnetite the pH_PZC_ of modified mesoporous silica changed to 8 which generated positive silica surface^43^.

The adsorption experiments under present research were done at pH 7. At this pH, surface of pure MOSF can be regarded negative charged. As result, adsorption capacity of pure MOSF was very much low [(0.58 and 2.49 mg/g for As(III) and As(V) at 1–25 mg/l initial concentrations]. Conversely, pure MF_0.33_ had pH_PZC_ 10.25 which indicated that MF_0.33_ surface showed higher amount of positive sites at pH 7 (pH ˂ pH_PZC_) which favors arsenic adsorption [(103.94 and 45.52 mg/g for As(III) and As(V)]. After the impregnation of MF_0.33_ in MOSF, pH_PZC_ was changed to 9.02 and adsorption capacity was 42.80 and 39.73 mg/g for As(III) and As(V) respectively. The EDS and elemental analysis data confirmed that MF_0.33_-MOSF contain less Fe and Mg than MF_0.33_, which was the main reason responsible for the lower arsenic adsorption capacity. At pH 7, Arsenous acid (H_3_AsO_3_) is the available form of As(III) and H_2_AsO_4_^-^ and HAsO_4_^2−^ are two available forms of As(V) in aqueous solution. So, the MF_0.33_ and MF_0.33_-MOSF nanomaterials having positively charged surface might be easily attached the negatively charged As(V) through complex formation, ion exchange and electrostatic attraction. The possible reactions are:

H_3_AsO_3_ species of As(III) has no charge which can adsorbed through complex formation. The possible adsorption reactions of As(III) are:

$${\text{MF}}_{{\text{x}}} {\text{ - OH}}_{{({\text{surface}})}} + {\text{ H}}_{{3}} {\text{AsO}}_{{3}} \leftrightarrow {\text{ MF - H}}_{{2}} {\text{AsO}}_{{3}} + {\text{ H}}_{{2}} {\text{O }}\left[ {{\text{Inner - sphere }}\;{\text{surface }}\;{\text{complex}}\;{\text{ formation}}} \right]$$

$${\text{MF}}_{{\text{x}}} {\text{ - OH}}_{{({\text{surface}})}} + {\text{ 3H}}^{ + } + {\text{ AsO}}_{{3}}^{{{3} - }} \leftrightarrow {\text{ MF}}_{{\text{x}}} {\text{ - OH}}_{{2}} {\text{ - AsO}}_{{3}} \left[ {{\text{Outer - sphere}}\;{\text{ surface }}\;{\text{complex}}\;{\text{ formation}}} \right]$$

Possible reactions for H_2_AsO_4_^-^ and HAsO_4_^2-^ adsorption through chemisorption

$${\text{MF}}_{{\text{x}}} {\text{ - OH}}_{{({\text{surface}})}} + {\text{ H}}^{ + }_{{({\text{aq}})}} \leftrightarrow {\text{ MF}}_{{\text{x}}} \;{\text{OH}}_{{2}}^{ + } \left[ {{\text{at }}\;{\text{pH}}\; < \;{\text{pH}}_{{{\text{PZC}}}} } \right]$$

$${\text{MF}}_{{\text{x}}} {\text{ - OH}}_{{2}}^{ + } + {\text{ H}}_{{2}} {\text{AsO}}_{{4}}^{ - } \leftrightarrow {\text{ MF}}_{{\text{x}}} {\text{ - OH}}_{{2}} {\text{ - H}}_{{2}} {\text{AsO}}_{{4}} \left[ {{\text{through }}\;{\text{electrostatic}}\;{\text{ attraction}}} \right]$$

$${\text{MF}}_{{\text{x}}} {\text{ - OH}}_{{({\text{surface}})}} + {\text{ H}}_{{2}} {\text{AsO}}_{{4}}^{ - } \leftrightarrow {\text{ MF - HAsO}}_{{4}} + {\text{ H}}_{{2}} {\text{O }}\left[ {{\text{Inner - sphere }}\;{\text{surface}}\;{\text{ complex}}\;{\text{ formation}}} \right]$$

$${\text{MF}}_{{\text{x}}} {\text{ - OH}}_{{({\text{surface}})}} + {\text{ H}}^{ + } + {\text{ HAsO}}_{{4}}^{ - } \leftrightarrow {\text{ MF}}_{{\text{x}}} {\text{ - OH}}_{{2}} {\text{ - HAsO}}_{{4}} \left[ {{\text{Outer - sphere }}\;{\text{surface }}\;{\text{complex}}\;{\text{ formation}}} \right]$$

Considering above reactions, chemisorption was the dominant adsorption mechanism for As(III) and As(V) adsorption. Chemisorption process occurred through complex formation, ion exchange and electrostatic attraction. The literature data regarding arsenic oxyanions adsorption on spinal ferrites revealed that the dominant adsorption mechanism was surface complex formation^1,18,38,44–46^.

#### Comparison with other adsorbents for arsenic adsorption

For the first time, magnesium ferrite was impregnated in MOSF which showed enhanced surface area, mesoporous pore size and high pore volume. The material was applied as adsorbent for arsenic removal at pH 7 and 25 °C. The maximum As(III) and As(V) adsorption capacity were found 42.80 and 39.73 mg/g respectively. The material adsorbed higher amount of As(III) and As(V) from water compared to other adsorbents listed in Table S5 at pH 7.

Fe modified activated carbon material showed lower As(III) and As(V) adsorption capacity^4,47^. In addition, longer equilibrium time and membrane filtration with N_2_ purging applied for adsorption which limited the research for further application in water treatment. Gupta and Ghosh^48^ synthesized Fe(III)-Ti(IV) binary oxide which removed 85 mg/g As(III) within 4.5 h at pH 7. However, the material removed very much lower amount (14 mg/g) of As(V) within 7.5 h at pH 7. In addition, they maintained pH for certain time period until equilibrium reached during adsorption. Furthermore, membrane filtration was applied for separation of adsorbent from water^48^. Fe_3_O_4_-MnO_2_ binary oxide adsorbed 32.13 mg/g As(V) at pH 5^49^. Yu et al.^6^ synthesized cellulose@Fe_2_O_3_ magnetic composites and applied for arsenic removal. The nanocomposites adsorbed 23.16 mg/g As(III) at pH 7.5 and 32.11 mg/g As(V) at pH 2. 3D organized mesoporous silica coated with Fe and Al oxide was synthesized by Glocheux et al.^50^ which adsorbed 55 mg/g As(V) at pH 5 and 35 mg/g As(V) at pH 4 respectively. Fe_2_O_3_/SiO_2_ nanocomposite adsorbed only 21.50 mg/g As(III) at pH 7.5 and 14.90 mg/g As(V) at pH 4.5. In both cases, adsorption temperature was 35 °C^51^. Ahangari et al.^52^ synthesized nickel-zinc ferrite modified carbon nanotubes nanocomposite for As(V) removal from wastewater. The nickel-zinc ferrite (NZF) and carbon nanotube nickel-zinc ferrite (CNZF) nanocomposites adsorbed 56 and 66 mg/g As(V) at pH 2 and 6 g/l of adsorbent dosage^52^. Therefore, comparing with activated carbon, carbon nanotube, cellulose, binary oxides and other silica modified nanomaterial/nanocomposites, MF_0.33_-MOSF found efficient adsorbent material for As(III) and As(V) removal from water.

Furthermore, magnesium ferrite impregnated MOSF was synthesized following MF_0.33_ synthesis process where MOSF added with precursor materials of MF_0.33_ therefore, no further synthesis process had been applied. For synthesis of other modified materials (Table S3), complex synthesis process were involved which needed higher energy, time and cost. Therefore, based on synthesis, characteristics and adsorption capacity, MF_0.33_-MOSF nanomaterial was considered as one of an ideal material for water treatment. This nanomaterial might work as a green material for sustainable water treatment technology development in future.

## Conclusion

Iron based nanomaterials have drawn much attention in adsorptive removal of pollutants from water because of their availability, properties, adsorption capacity, lower cost and ecofriendly nature. Among them, magnesium ferrite nanomaterials showed greater properties and performance in water treatment process. However, being a nanomaterial, it has limitations such as stability, aggregate formation and separation. Therefore, modification of magnesium ferrite nanomaterials with well-structured materials having higher stability, surface area and easy separation from solution are very much popular in recent time. Following that, a new combination, MF_0.33_-MOSF has been successfully synthesized and applied for As(III) and As(V) removal from water at pH 7. MOSF having negatively charged surface area at pH 7 showed repulsion against arsenic oxyanions. However, adsorption properties were increased after impregnation with MF_0.33_. The magnetic MF_0.33_-MOSF showed better morphological properties and adsorption capacity (at pH 7) compared to other nanomaterials found in literature. Adsorption results best fitted with Freundlich isotherm and adsorption mechanism was found to chemical sorption through complex formation and electrostatic attraction on heterogeneous surface. Modification of MF_0.33_ with MOSF using simple, less time-consuming and cost-effective procedure was successfully confirmed to be effective. Therefore, magnetic MF_0.33_-MOSF composites could be competitive nanoadsorbents for arsenic removal from water.

### Supplementary Information

Supplementary Tables.

## Data Availability

Research data and materials are available upon request from the corresponding author and following journal guideline.
