# Supplementary material for: Facile synthesis of flower shaped magnesium ferrite (MgFe2O4) impregnated mesoporous ordered silica foam and application for arsenic removal from water
Source: Sci Rep. 2023 Dec 7;13:21617. doi: 10.1038/s41598-023-48327-7 (PMC10703909; doi:10.1038/s41598-023-48327-7)
Supplement: Supplementary file 1 — Supplementary Tables. [file 41598_2023_48327_MOESM1_ESM.docx]

**Supplementary materials**

**Facile synthesis of flower shaped magnesium ferrite (MgFe_2_O_4_) impregnated mesoporous ordered silica foam and application for arsenic removal from water**

Md. Jamal Uddin^1^* and Yeon-Koo Jeong^2^

1. * Corresponding author: Department of Soil and Environmental Sciences, University of Barisal, Barisal, Bangladesh.

2. Department of Environmental Engineering, Kumoh National Institute of Technology, 61 Daehak-ro, Gumi, Gyeongbuk 39177, Republic of Korea

Contact No: +8801723108347, E-mail: jamal_swedu@yahoo.com

**Table S1** Morphological properties of MF_0.33_, MOSF and MF_0.33_-MOSF nanomaterials.

| **Material** | **BET surface area (m^2^/g)** | **Pore size (nm)** | **Pore volume (cm^3^/g)** | **Average particle size (nm)** |
| --- | --- | --- | --- | --- |
| MF_0.33_ | 200.3633 | 4.17 | 0.208315 | 29.9456 |
| MOSF | 412.8638 | 11.41 | 0.801195 | 14.5326 |
| MF_0.33_-MOSF | 427.0448 | 6.05 | 0.468433 | 14.0500 |

**Table S2** Magnetic properties of MF_0.33_ and MF_0.33_-MOSF.

| **Property** | **MF_0.33_** | **MF_0.33_-MOSF** |
| --- | --- | --- |
| Ms (emu/g) | 16.90 | 1.36 |
| Mr (emu/g) | 1.30 | 0.14 |
| Mr/Ms | 0.08 | 0.10 |
| Hc (Oe) | 30.15 | 153.14 |

**Table S3** Comparison of morphological properties of magnesium ferrite nanomaterials.

| **Material** | **Pore size**  **(nm)** | **Pore volume**  **(cm^3^/g)** | **Surface area**  **(m^2^/g)** | **Synthesis method** | **Ref.** |
| --- | --- | --- | --- | --- | --- |
| MgAlNi-BaFe_2_O_4_ | 5.48 | 0.32 | 132.38 | Co-precipitation | [31] |
| MgFe_2_O_4_-NH_2_ | 1.5-28.4 | 0.30 | 47.00 | Co-precipitation | [32] |
| MgFe_2_O_4_-OH | 1.5-11.8 | 0.40 | 79.00 | Hydrothermal |  |
| MgFe_2_O_4_@SiO_2_ | 6 | 0.10 | 20.00 | Refluxing | [30] |
| MgFe_2_O_4_/rGO | 4.20 | - | 35.40 | Sol-gel & Hummer | [33] |
| MgFe_2_O_4_/graphene | 2-300 | - | 47.15 | Solvothermal | [34] |
| MgFe_2_O_4_/g-C_3_N_4_-10 | 5-15 | - | 16.80 | Solvothermal | [35] |
| GO-loaded- MgFe_2_O_4_ | - |  | 114.59 | Hummer & Solvothermal | [36] |
| GO-coated- MgFe_2_O_4_ | - | - | 117.83 | Hummer & Solvothermal |  |
| MgFe_2_O_4_/γ-Fe_2_O_3_ | 1.89 | 0.357 | 68.20 | Template-hydrothermal | [37] |
| MgFe_2_O_4_@SiO_2_ | 5.55 | 0.22 | 102.20 | Sol-gel | [38] |
| SiO_2_@MgFe_2_O_4_ | 3.54 | 0.68 | 459.10 |  |  |
| **MF_0.33_-MOSF** | **6.05** | **0.47** | **427.04** | **Solvothermal** | **Present study** |

**Table S4** Equilibrium isotherm for As(III) and As(V) adsorption on MF_0.33_-MOSF nanomaterial.

| **Adsorbate** | **Synthesized nanoadsorbents** | **Freundlich** | | | **Langmuir** | | |
| --- | --- | --- | --- | --- | --- | --- | --- |
|  |  | **K_F_** | **n** | **r^2^** | **q_m_** | **K_L_** | **r^2^** |
| As(III) | MF_0.33_ | 16.912 | 2.897 | 0.985 | 103.94 | 0.052 | 0.967 |
|  | MF_0.33_-MOSF | 4.770 | 2.508 | 0.982 | 42.80 | 0.030 | 0.976 |
| As(V) | MF_0.33_ | 10.242 | 3.147 | 0.985 | 45.52 | 0.111 | 0.937 |
|  | MF_0.33_-MOSF | 1.097 | 1.591 | 0.998 | 39.73 | 0.010 | 0.983 |

**Table S5** Comparison on arsenic adsorption capacity with different adsorbents.

| **Adsorbate** | **Adsorbent** | **Special requirements during adsorption process** | **Equilibrium time (hour)** | **pH** | **Maximum adsorption capacity (mg/g)** | **Ref.** |
| --- | --- | --- | --- | --- | --- | --- |
| As(III) | NZVI/AC | 0.2 μm membrane filtration & Purging with N_2_ | 72 | 6.5 | 18.20 | [4] |
|  | Fe_3_O_4_-MnO_2_ | - | 24 | 5 | 72.83 | [50] |
|  | Fe(III)-Ti(IV) binary oxide | 0.45 μm membrane filter &  pH was maintained during the adsorption period until reached equilibrium | 4.5 | 7 | 85.00 | [51] |
|  | Fe_2_O_3_/SiO_2_ | Temperature 35 ˚C | 24 | 7.5 | 21.50 | [52] |
|  | Cellulose@Fe_2_O_3_ | - | - | 7.5 | 23.16 | [6] |
|  | **MF_0.33_-MOSF** | **Simple adsorption process** | **12** | **7** | **42.80** | **Present study** |
| As(V) | Fe modified-AC | - | 48 | 7 | 35.34 | [53] |
|  | NZVI/AC | 0.2 μm membrane filtration & Purging with N_2_ | 72 | 6.5 | 12.00 | [4] |
|  | Fe_3_O_4_-MnO_2_ | - | 24 | 5 | 32.13 | [50] |
|  | Fe(III)-Ti(IV) binary oxide | 0.45 μm membrane filter &  pH was maintained during the adsorption period until reached equilibrium | 7.5 | 7 | 14.00 | [51] |
|  | Fe_2_O_3_/SiO_2_ | Temperature 35 ˚C | 24 | 4.5 | 14.90 | [52] |
|  | Cellulose@Fe_2_O_3_ | - | - | 2 | 32.11 | [6] |
|  | 3D OMS coated Fe-Al oxide | - | 48 | 5 | 55.00 | [54] |
|  |  |  |  | 4 | 35.00 |  |
|  | NZF | 6 g/L adsorbent dose | 30 min | 2 | 56.00 | [55] |
|  | NZF/CNT |  |  |  | 66.00 |  |
|  | **MF_0.33_-MOSF** | **Simple adsorption process** | **12** | **7** | **39.73** | **Present study** |

**Note:** AC = Activated carbon; NZVI = Nano zero-valent iron; OMS = Organized mesoporous silica; NZF = Nickel-zinc ferrite; CNT = Carbon nanotube.
